# Characterization of folic acid‐functionalized PLA–PEG nanomicelle to deliver Letrozole: A nanoinformatics study

**DOI:** 10.1049/nbt2.12073

**Published:** 2021-11-23

**Authors:** Neda Rostami, Reza Davarnejad

**Affiliations:** ^1^ Department of Chemical Engineering, Faculty of Engineering Arak University Arak Iran

**Keywords:** cancer, cell membrane, Letrozole, molecular dynamics, nanomicelle, targeted delivery

## Abstract

Effective cancer treatment is currently the number one challenge to human health. To date, several treatment methods have been introduced for cancer cell targeting. Among the proposed new methods for attacking cancer cells, nanotechnology has attracted much attention. Hence, various nanocarriers have been developed for targeted delivery of available drugs and improve their effectiveness against malignant cells. The PLA–PEG functionalised with folic acid (PLA–PEG‐FA) is one of the nanocarriers with a limited range of applications for targeting cancer cells. In this investigation, different types of in‐silico methods such as molecular docking approach, molecular dynamics simulation and free energy calculations are employed to characterise the carriers studied. The effectiveness of PLA–PEG‐FA and PLA–PEG in delivering Letrozole as an aromatase inhibitor in cancer cells is examined. It is found that in the presence of folic acid, the stability and cell membrane permeability of nanomicelle are increased. Therefore, PLA–PEG‐FA can be considered as a versatile carrier that can increase the effectiveness of aromatase inhibitors (such as Letrozole) and reduce their side effects.

## 1 INTRODUCTION

There is no doubt that cancer should be regarded as one of the biggest health problems faced by a person, and billions of dollars are spent on prevention and treatment every year [1]. In view of the importance of this topic, researchers from various scientific fields have participated in the design and development of new strategies for targeted cancer therapy [2]. Although great efforts have been made for cancer therapy, due to the biological complexity of the pathways involved in these abnormal physiological conditions, no method has been proposed to completely remove malignant cells from patients [3]. According to the World Health Organization (WHO) reports, cancer after the cardiovascular disease is the second leading cause of death in developed and underdeveloped countries [4]. Based on the American Cancer Society estimates in 2021, the numbers of new cancer cases and deaths in the United States were 1,898,160 and 608,570, respectively [5].

Of the 210 cancers, breast cancer in women and lung cancer in men are the most common cancers, while in both sexes, lung cancer is the deadliest cancer [6]. Among breast cancer treatments, hormone therapy often given as an adjuvant therapy, is one of the main therapeutic strategies, and in this regard, a variety of therapeutics have been developed for this purpose [7]. Inhibitors that block the hormone binding to the cell surface receptors (Tamoxifen and Toremifene), drugs that reduce ovarian function (Goserelin) and medications that inhibit oestrogen expression after menopause (aromatase inhibitors) are some of the most well‐known drugs used in hormone therapy [8].

As mentioned, the inhibition of oestrogen function and related biological pathways is an important part of cancer hormone therapy. Aromatase inhibitors, by inhibiting the aromatisation process that converts androgens to oestrogens, decrease the chance of cancer progression [9]. Although oestrogen is expressed and secreted from the ovaries in premenopausal women, in postmenopausal women it is mainly expressed in other parts of the body such as breast adipocytes which can affect in situ cancer cells that are oestrogen receptor‐positive and with this trick increase the risk of cancer recurrence [10]. However, the use of this class of drugs before menopause leads to an opposite pharmacological performance by activating the secretion of gonadotropin from the hypothalamus and pituitary axis which leads to increased synthesis and secretion of androgens [11]. Aromatase inhibitors are divided into two categories that include irreversible steroidal inhibitors (Exemestane) and non‐steroidal inhibitors (Anastrozole and Letrozole). The most common side effects of this class of medications are infertility, skeletal system disorders, aggressive demeanour, adrenal‐kidney disorders, liver dysfunction and alopecia [12].

Among aromatase inhibitors, Letrozole is a more widely used drug that is of great importance in the medication regimen of most patients that carries oestrogen receptor‐positive cancer cells [13]. Nevertheless, side effects that occurred following off‐target activity and low efficacy are some of the negative aspects of Letrozole that drug companies are trying to address [14]. In recent years, by employing nanotechnology and targeted drug delivery strategies mainly for purposes such as drug release optimisation, reducing required concentration, increasing half‐life and drug efficiency, successful experiences have been achieved in improving the pharmacological attributes of Letrozole [15]. To date, many investigations have been conducted to design nanoparticles for targeted delivery of Letrozole and other aromatase inhibitors, which in a significant number of polymeric hybrid nanomicelles (NMs) were utilised due to some advantages such as affordability, biocompatibility and high biodegradability [16, 17]. Synthetic materials namely PLA–PEG, PEG–PAA, PEG‐b‐PCL and PEG–PEI are the most well‐known carriers of this category [18, 19]. Besides, in many cases for the promotion of NMs targeted therapy potency, chemical fusion partners are added to the designed carriers. The folic acid receptor is a versatile biomarker for cancer cell targeting since its overexpression on different types of tumours. Hence, in targeted therapy approaches folic acid has extensively been exploited to promote the performance of developed drugs against malignant cells [20].

Given that breast cancer cells are highly greedy for the uptake of folic acid, employing this vitamin in targeted delivery is very popular [21, 22]. Also, poly‐(lactic acid) (PLA) and poly‐(ethylene glycol) (PEG) are widely employed in drug delivery investigations due to their advantages such as affordability, easy synthesis, biodegradability and low toxicity [23, 24]. The PLA–PEG and PLA–PEG‐FA copolymers were used as a nanocarrier to deliver various drugs such as doxorubicin and docetaxel [25], however, the molecular aspects and their ability to interact with the cell membrane are overlooked by researchers. Furthermore, the potency of these copolymers has not yet been used to deliver aromatase inhibitors. The present study aims to investigate the molecular behaviour of Letrozole/PLA−PEG complex compared with that of Letrozole/PLA−PEG‐FA. In this investigation, molecular docking, molecular dynamics (MD) and free energy calculation are applied to investigate the dynamic and thermodynamic behaviours of Letrozole/PLA−PEG and Letrozole/PLA−PEG‐FA with di‐palmitoyl‐phosphatidyl‐choline (DPPC) membrane at the atomic level.

## 2 METHODOLOGY

### 2.1 Inputs and preparations

To design the NMs, desired materials for our study including Letrozole (PubChem ID 3902), PLA (PubChem ID 612) and folic acid (PubChem ID 135398658) were retrieved in SDF format from the PubChem [26, 27]. All structures were optimised via ChemDraw (*v19.0*) for NMs designing and other chemical modifications. In the next step, the bilayer membrane 3D structure was created using the CHARMM‐GUI server, which is an online server owned by the LEHIGH University [28]. This server is a versatile tool for atomic‐level parameterisation of different types of biomolecules and small molecules. In the present study, the DPPC membrane which is frequently used in biological studies was employed to investigate the behaviour of NMs in the vicinity of the membrane. In this step, by utilising the membrane builder option of CHARMM‐GUI, the DPPC membrane with desired attributes was built with membrane preparation box type set to Rectangular. The length of the *Z*‐axis was adjusted based on the hydration number (33 water molecules per one lipid molecule), while the length of *XY* was set to numbers of lipid components (*XY* dimension ratio: 1). The number of lipid components was set to 64 particles for upper and lower layers (overall 128 DPPC molecules). Moreover, value 63 Å was chosen for the surface area [29]. In the ionisation step, 0.15 M NaCl (ion concentration) was added to the system using the Monte‐Carlo algorithm. In addition, in the last step of membrane preparation, force field CHARMM36 was chosen for membrane parametrisation [30].

### 2.2 Docking methodology

In general terms, local and global docking are two versatile algorithms for studying interactions between molecules and predicting the binding affinities [31]. In this study, docking was performed to investigate the interaction mode of Letrozole with the designed NMs, calculate the binding energy and propose the most stable complex. Before docking, nano‐systems were prepared using Autodock tools (*v1.5.6*). For this purpose, charges (Gasteiger algorithm) and polar hydrogens were assigned for complexes. In order to execute the docking of NMs with Letrozole, AutoDock Vina (an open‐source tool) was utilised [32]. During interactions modelling, the desired grid box for designed NMs was generated with 40 × 40 × 40 Å dimensions and value 0.357 Å was considered for spacing. For docking energies exploration, the genetic algorithm (GA) was chosen. Furthermore, GA runs and population size were set at 10 and 150, respectively. Visualisation of interactions was carried out by the discovery studio visualiser (*v2021*).

### 2.3 Molecular dynamics simulation

Molecular dynamics also known as the computational molecular microscope is an approach for studying biological systems at the atomic scale [33]. Here, MD was implemented to evaluate the extent of water and NMs’ penetration into the membrane, membrane disruption and interaction of designed nanostructures with membranes in an environment similar to the physiological condition. The MD simulation process was accomplished by GROMACS (*v2021*) in Ubuntu (*20.04 LTS*) [34]. The topology file of the membrane was generated using CHARMM‐GUI (CHARMM36 force field), while Swiss‐PARAM which is compatible with the CHARMM all atoms force field was used for Letrozole and NMs parametrization [35]. Prior to MD, designed NMs were placed within 5 Å distance of the membrane. In equilibration steps, the canonical ensemble (NVT) was performed for temperature coupling using the V‐rescale algorithm at a constant temperature of 310 K for 500 ps. Additionally, the isothermal–isobaric ensemble (NPT) at a constant pressure of 1 bar was applied for 5 ns using the Nose‐Hoover algorithm [36]. In the production step, all complexes were simulated for 200 ns. Throughout MD, constraints were applied for all bonds by employing the LINCS method. In the last step, some functions such as root‐mean‐square deviation (RMSD), radial distribution function (RDF), root‐mean‐square fluctuation (RMSF), solvent accessible surface area (SASA), 2D‐density, Mindist and mean square displacement (MSD) were extracted from the output trajectory files.

### 2.4 Free energy investigation

The molecular mechanics–Poisson Boltzmann surface area (MM–PBSA) function is a widely used approach in the investigation of free energy to determine the stability of systems [37]. The g_mmpbsa module of the GROMACS which is equipped with the MM–PBSA algorithm is a capable tool for calculating free energy by analysing the MD trajectory files. In this tool, the total free energy of the system is determined by calculating the energy terms such as solvation free energy (Gpolar + Gnonpolar) and molecular mechanics potential energy (EM bonded + (EvdW + Eelec)) [38]. Regarding the purpose of the current study, the output trajectory files were conducted to the free energy calculation process for further evaluation. During the calculation of free energy, the SASA model was utilised to calculate the non‐polar solvation energy, so the pdie option was set to 2. Furthermore, the MmPbSaStat.py script was used to calculate the binding energy.

## 3 RESULTS AND DISCUSSION

### 3.1 Nanomicelles, drug and membrane preparation

Letrozole and other compounds retrieved from PubChem and for preparation were conducted to ChemDraw. NMs designing was done based on previous studies [25]. The structural characteristics of the compounds studied are listed in Table 1.

**TABLE 1:** Some of the molecular characteristics for input structures

Compound	MW (g/mol)	TPSA (Å^2^)	Log *P*	Heavy atoms	Synthetic accessibility
Letrozole	285.30	78.29	2.32	22	2.58
PLA–PEG	134.13	66.76	−0.39	9	2.04
PLA–PEG‐FA	572.40	288.12	−1.16	42	3.47

Abbreviations: MW, molecular weight; TPSA, topological polar surface area.

**FIGURE 1:**
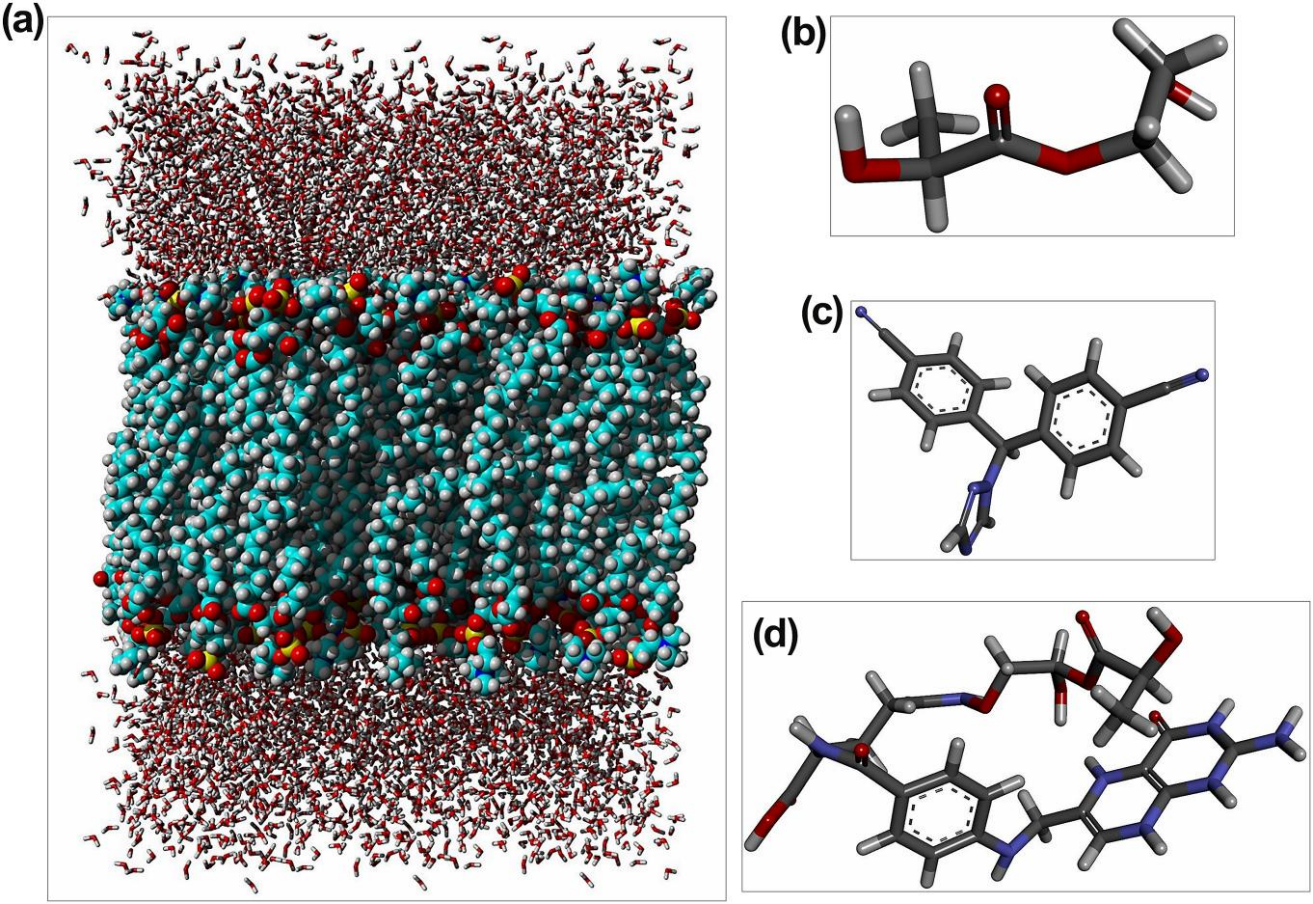
Graphical representation of input structures. (a) DPPC, (b) PLA–PEG, (c) Letrozole and (d) PLA–PEG‐FA

In addition, the structure of the prepared membrane and other desired compounds is shown in Figure 1. Membrane solvation was performed in the direction of the Z‐axis to speed up the simulation time. The designed membrane consisted of 128 DPPC molecules (64 molecules in the upper and lower layers), 4224 water molecules and 14 ions (7 Cl^−^ AND 7 Na^+^). The whole number of atoms in the membrane system was 29,326, and the size of the *X*, *Y* and *Z*‐axis were 63.49, 63.49 and 77.82 Å, respectively.

### 3.2 Docking

**FIGURE 2:**
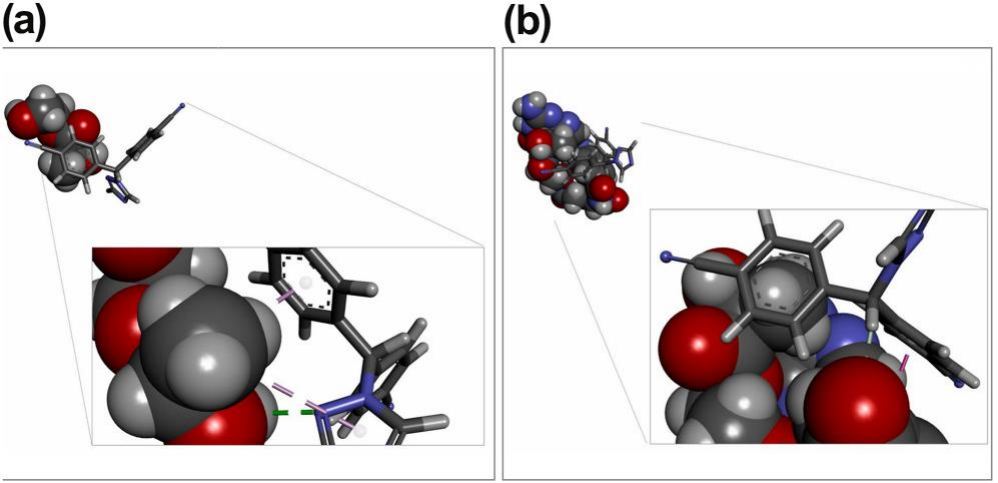
(a) Representation of Letrozole docked to PLA–PEG (−9.49 kcal/mol) and (b) Letrozole with PLA–PEG‐FA (−10.90 kcal/mol)

Molecular docking has been used to investigate interactions between Letrozole and designed NMs (PLA–PEG & PLA–PEG‐FA). In this step, examination of binding type and conformational energy computation are the main objectives. After docking, the highest binding affinity conformation (lowest docking score) of Letrozole among 38 poses for PLA–PEG and 50 poses for PLA–PEG‐FA were chosen for further analysis. We found that the presence of folic acid increased the binding affinity between Letrozole and PLA–PEG (−10.90 kcal/mol). While, for PLA–PEG, Letrozole interacted with a binding affinity of −9.49 kcal/mol. From Figure 2, we can observe that the triazole ring of letrozole contributes to stabilising the interaction of the selected drug with chosen NM systems. In the presence of folic acid, NM mainly forms Pi‐Pi and Pi‐Lone interactions at 5.18 and 2.95 Å distance, respectively. However, PLA–PEG has formed a hydrogen bond and Pi‐alkyl interaction at 2 and 4.82 Å distance.

In order to examine the structures more closely, binding energy decomposition was done to track the complexes' stability. For this purpose, we followed two criteria factors to determine the finest configuration of docked systems: the lowest magnitude of binding free energy (kcal/mol) and the smallest value of RMSD. As shown in Table 2, the PLA–PEG‐FA gained lower binding energy than the PLA–PEG. Based on these results, it can be claimed that the presence of folic acid increases the stability and better encapsulation of the Letrozole in the nanocarrier.

**TABLE 2:** Summary of functions calculated in the molecular docking process, the unit of all energy terms is kcal/mol

NM	Functions				
	Binding free energy	VdW	Ligand efficiency	ES	RMSD (Å)
PLA–PEG‐FA	−10.90	−3.97	−0.14	−2.01	1.032
PLA–PEG	−9.49	−2.78	−0.09	−1.05	2.011

Abbreviations: ES, electrostatic energy; NM, nanomicelle; RMSD, root‐mean‐square deviation; VdW, Van der Waals energy.

### 3.3 Molecular dynamics analysis

**FIGURE 3:**
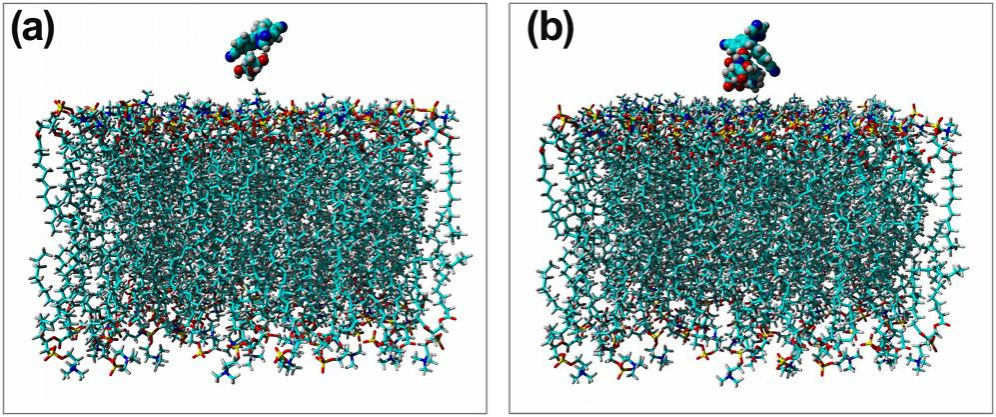
(a) Representation of Letrozole‐PLA–PEG‐DPPC membrane and (b) Letrozole‐PLA–PEG‐FA‐DPPC membrane

Throughout MD analysis different types of functions were employed for the examination of structural and molecular behaviours of designed NMs. The Letrozole‐NMs along with DPPC are presented in Figure 3, and these systems were subjected to MD simulation. In the first step of MD trajectory files analysis, RMSD and RMSF functions were employed to compare structural features of designed NMs. The RMSD which refers to the particles deviates the position from the reference position extracted for both designed NMs along with Letrozole. The slope of the RMSD graph indicates the conformational changes during the simulation. The closer the slope is to zero, the mean is the lower conformational changes. The average of RMSD for Letrozole in complex with PLA–PEG and PLA–PEG‐FA were 0.11 and 0.12 nm, respectively. Calculating the amount of RMSD for the NMs showed that the average of RMSD for PLA–PEG‐FA (0.458 nm) was higher than the PLA–PEG (0.0944 nm).

**FIGURE 4:**
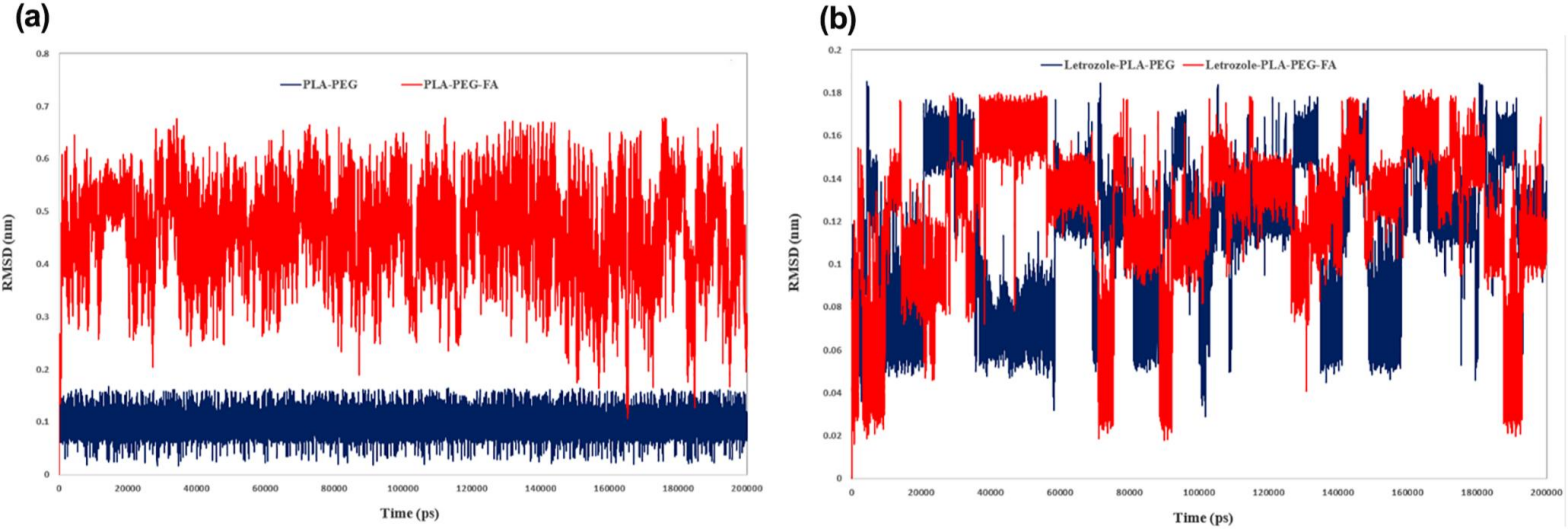
(a) RMSD values of nanomicelles throughout molecular dynamics and (b) RMSD values of Letrozole in both systems

The values of RMSD for Letrozole in both systems were almost the same, but the difference between the RMSD for NMs was significant, which could be due to the larger size of NM in the presence of folic acid (Figure 4). Indeed, the fusion of folic acid causes a widespread change in NMs’ conformation, which is expected to change the pharmacodynamics and pharmacokinetics characteristics of the nanocarrier.

**FIGURE 5:**
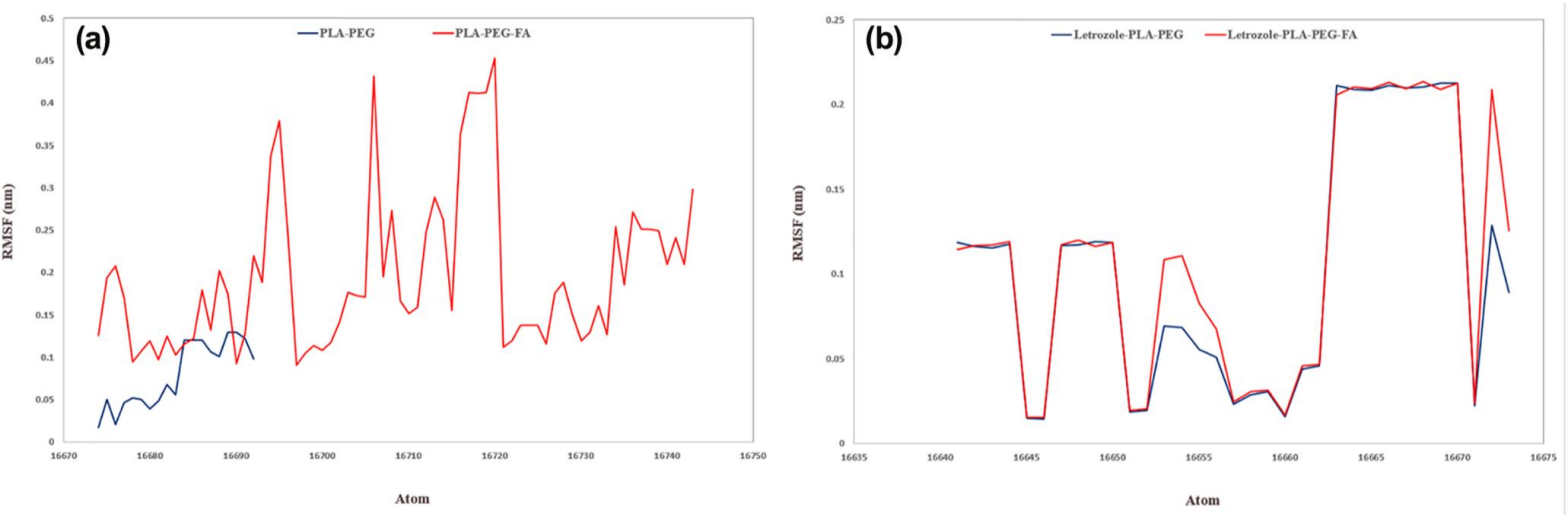
(a) RMSF degrees of nanomicelles during 200 ns molecular dynamics and (b) RMSF degrees of Letrozole in complex with designed NMS

RMSF was another function that was checked for both systems. Like RMSD, the average of Letrozole fluctuations was similar in both systems (PLA–PEG 0.10 nm and PLA–PEG‐FA 0.11 nm), while fluctuation examinations of NMs revealed a large difference in the average of the RMSF (PLA–PEG 0.08 nm and PLA–PEG‐FA 0.19 nm). The results of this step consistent with the RMSD output indicated an extensive change in the structural properties of NM due to the presence of folic acid (Figure 5). It is expectable that the large size of the folic acid will enhance the flexibility by increasing the available surface area of the nanomicelle facilitated trapping of Letrozole.

**FIGURE 6:**
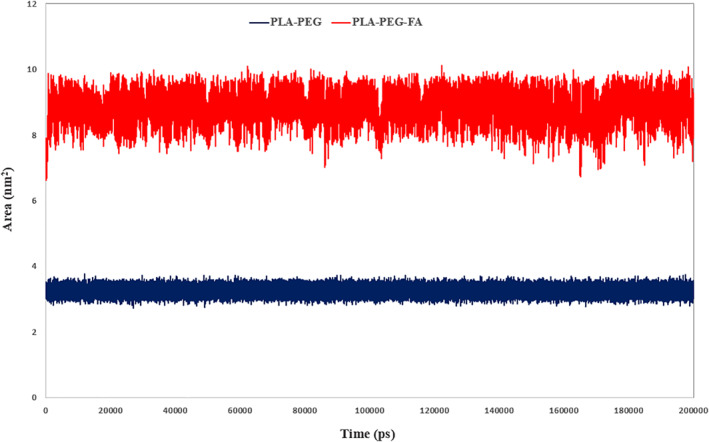
Schematic representation of solvent accessible surface area value for both systems, as can be seen, PLA–PEG‐FA has gained higher values

The second step of MD outputs’ analysis was performed to investigate the potency of desired NMs to the targeted delivery of Letrozole. With regard to the objective of this step five functions such as SASA, MSD, RDF, 2D‐density and Mindist were implemented. Given that Letrozole is a hydrophobic drug [39], the use of factors that promote its solubility can increase the performance of this drug. During the simulation and analysis of the SASA value for the studied NMs, we found that NM with folic acid has a much higher SASA value than another NM, so that it can improve the solubility of Letrozole more efficiently. We found that the hydrophilicity rate in the PLA–PEG‐FA is about three times more than PLA–PEG. The average of SASA for PLA–PEG and PLA–PEG‐FA were 3.25 and 8.81 nm^2^, respectively (Figure 6).

**FIGURE 7:**
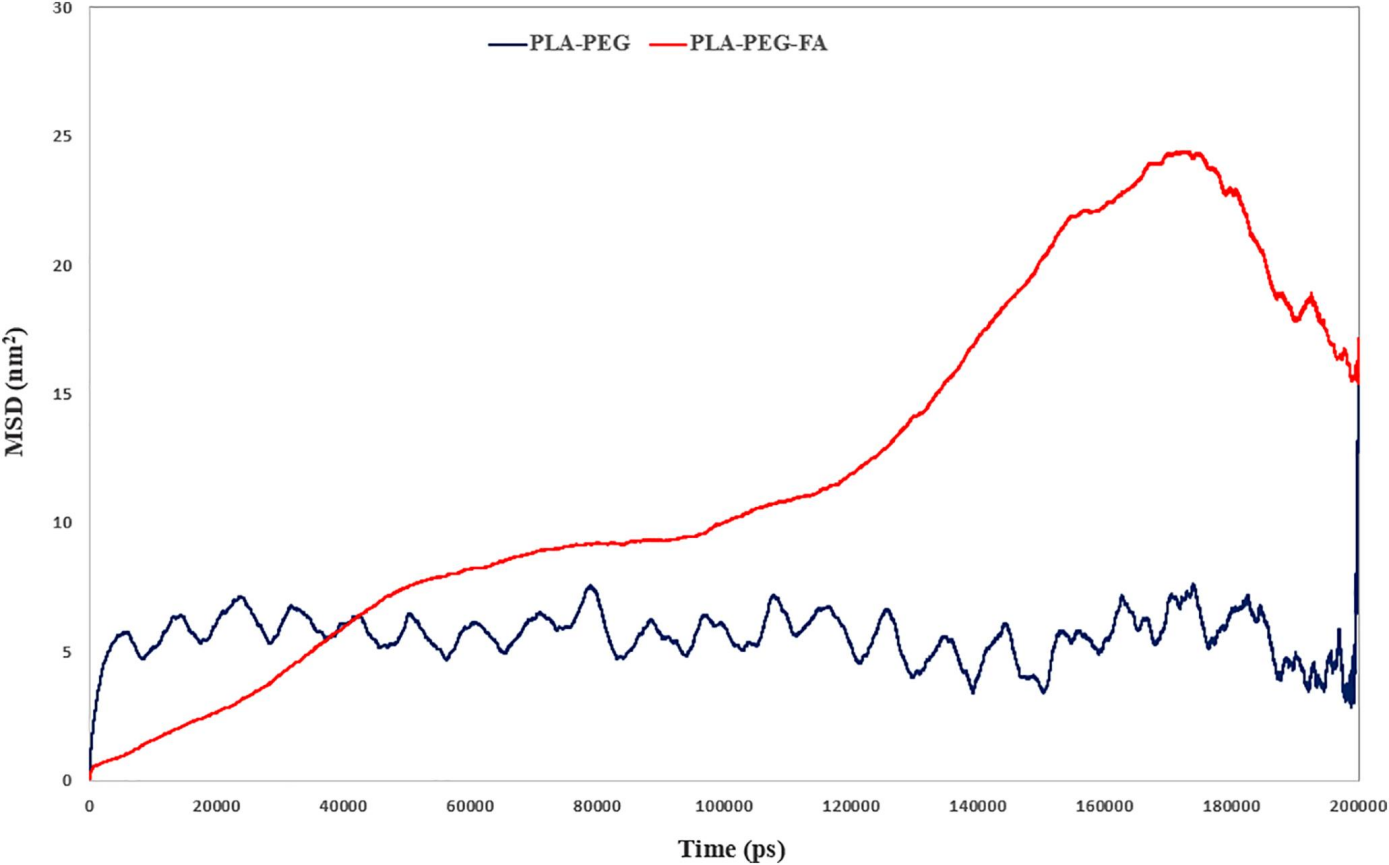
MSD diagram for PLA–PEG and PLA–PEG‐FA

In order to evaluate the penetration ability of the studied NMs in the DPPC membrane, the MSD function was calculated. Since in our case, MSD shows the amount of displacement in the *Z*‐axis [40], the average of this parameter for the PLA–PEG (5.61 nm^2^) and PLA–PEG‐FA (11.874 nm^2^) showed that the membrane diffusion of NM containing folic acid is much higher. The shape of the MSD graph also indicates that the PLA–PEG‐FA has reached a constant amount of penetration after 170 ns which means that the NM is trapped in the membrane, but for the PLA–PEG due to the lack of penetration the MSD is constantly oscillating (Figure 7). Due to Aromatase localised in the endoplasmic reticulum, promoting the entrance of letrozole into the PLA–PEG‐FA using cell can dramatically improve its function.

**FIGURE 8:**
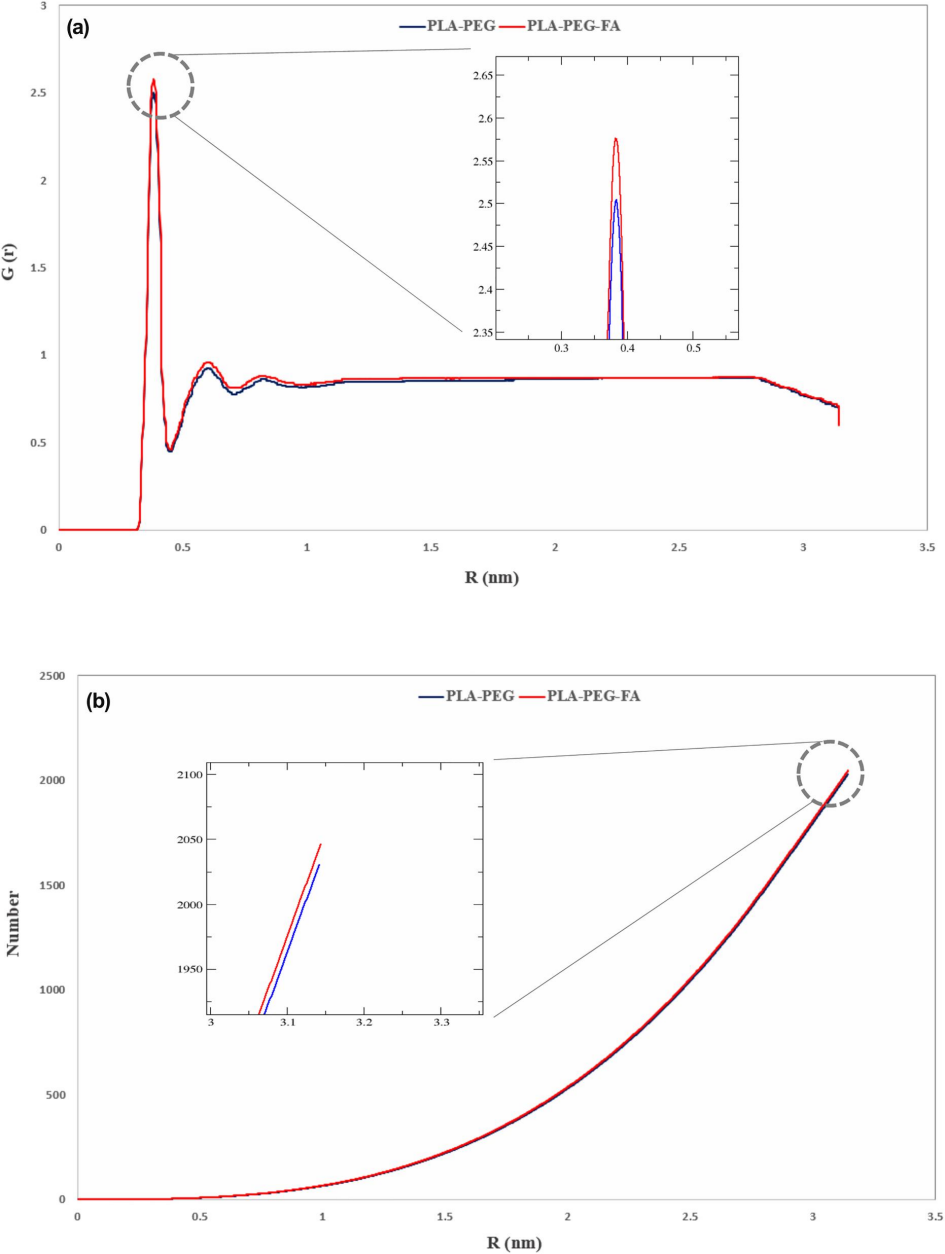
(a) RDF function for water molecules around phosphate groups of DPPC and (b) cumulative RDF for PLA–PEG and PLA–PEG‐FA

The RDF is another parameter that was investigated to examine the penetration capability of the target NMs [41]. The accumulation of water molecules around the DPPC was examined to achieve this purpose. Indeed, increasing the penetration of water into the membrane s a piece of evidence for membrane degradation. During RDF analysis, we found that in the PLA–PEG‐FA system the amount of water penetration into the membrane was higher than the PLA–PEG system. This result was consistent with the output of the MSD step and indicated the greater strength of the PLA–PEG‐FA for penetration into the membrane. The average RDF content for the PLA–PEG and PLA–PEG‐FA were 2.50 and 2.55, respectively. In addition, the cumulative RDF [42] showed that the number of water molecules that have penetrated the membrane (Figure 8) throughout the MD in the PLA–PEG‐FA system (2047 water molecules) was higher than that in PLA–PEG (2030 water molecules).

**FIGURE 9:**
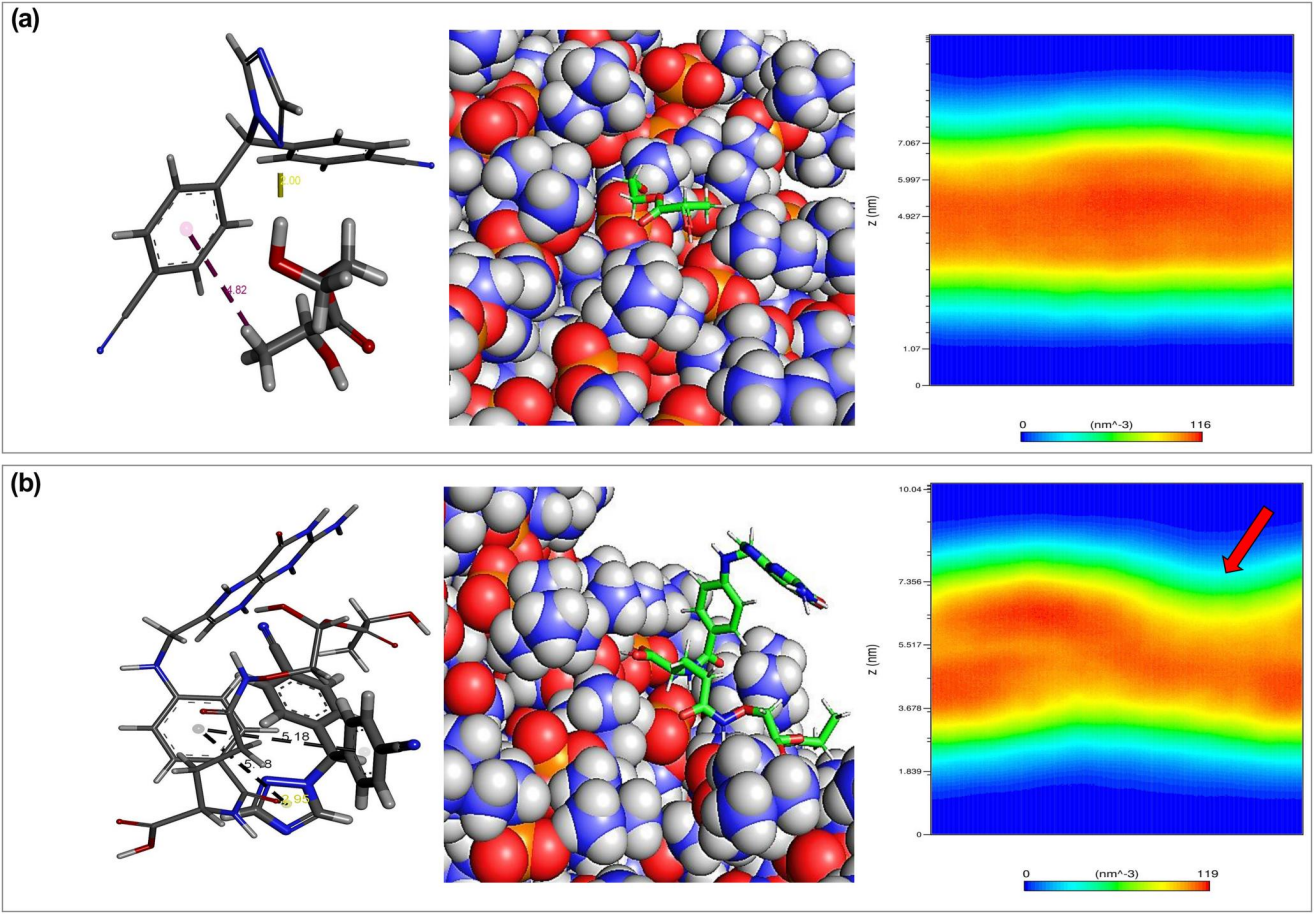
Representation of membrane degradation using the 2D‐density function. (a) PLA–PEG and (b) PLA–PEG‐FA

In continuation, the 2D‐density analysis was used for the examination of membrane degradation by the designed NMs. The 2D‐density showed that the rate of membrane degradation in the PLA–PEG‐FA‐treated system was much higher than the PLA–PEG (Figure 9). Better penetration into the membrane and the formation of a more stable complex with the studied drug were two critical factors for verifying the greater ability of the PLA–PEG‐FA compared with the PLA–PEG in the targeted drug delivery of Letrozole.

**FIGURE 10:**
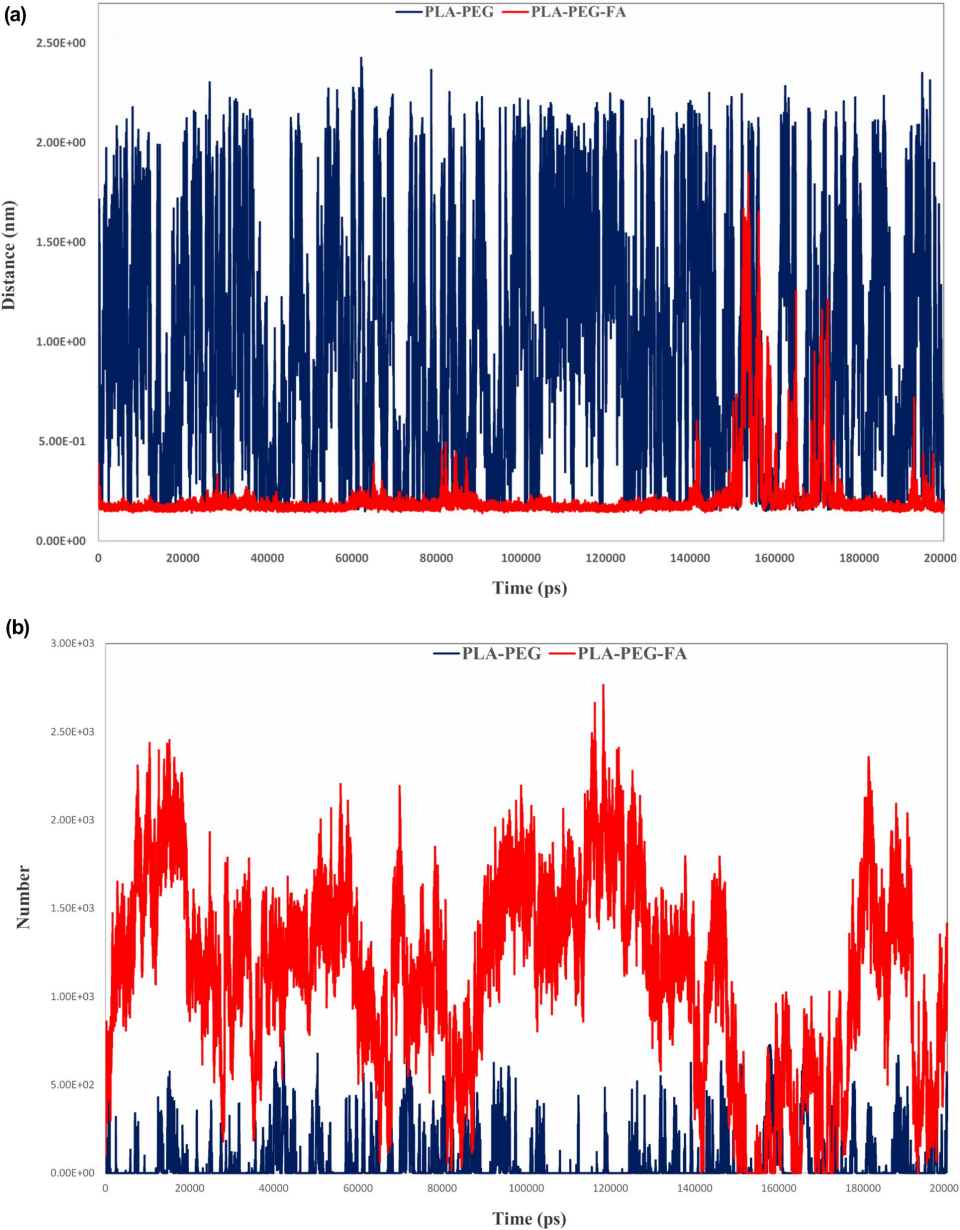
Mindist function output. (a) Minimum distance for studied nanomicelles and (b) the number of contacts at <0.6 nm for both systems

In the last step of MD analysis using the Mindist function [43], the number of contacts (<0.6 nm) between NMs and DPPC membrane plus the minimum distance were computed for components of the simulation. The number of interactions was 1124 for the DPPC & PLA–PEG‐FA complex and 58 for the DPPC & PLA–PEG. Also, the average of minimum distance was 0.2 nm for the DPPC & PLA–PEG‐FA system and 0.94 nm for the DPPC & PLA–PEG (Figure 10).

Shorter distance and higher interaction potency observed during Mindist analysis were two key confirmations for better trapping of PLA–PEG‐FA into the membrane than another NM. The findings of the second step of MD analysis, which was performed to evaluate the effectiveness of designed NMs in the targeted delivery of Letrozole were consistent with the observations during docking analysis. In all steps, we observed that PLA–PEG‐FA had higher membrane penetration and Letrozole targeted delivery potency.

### 3.4 Free energy analysis

**FIGURE 11:**
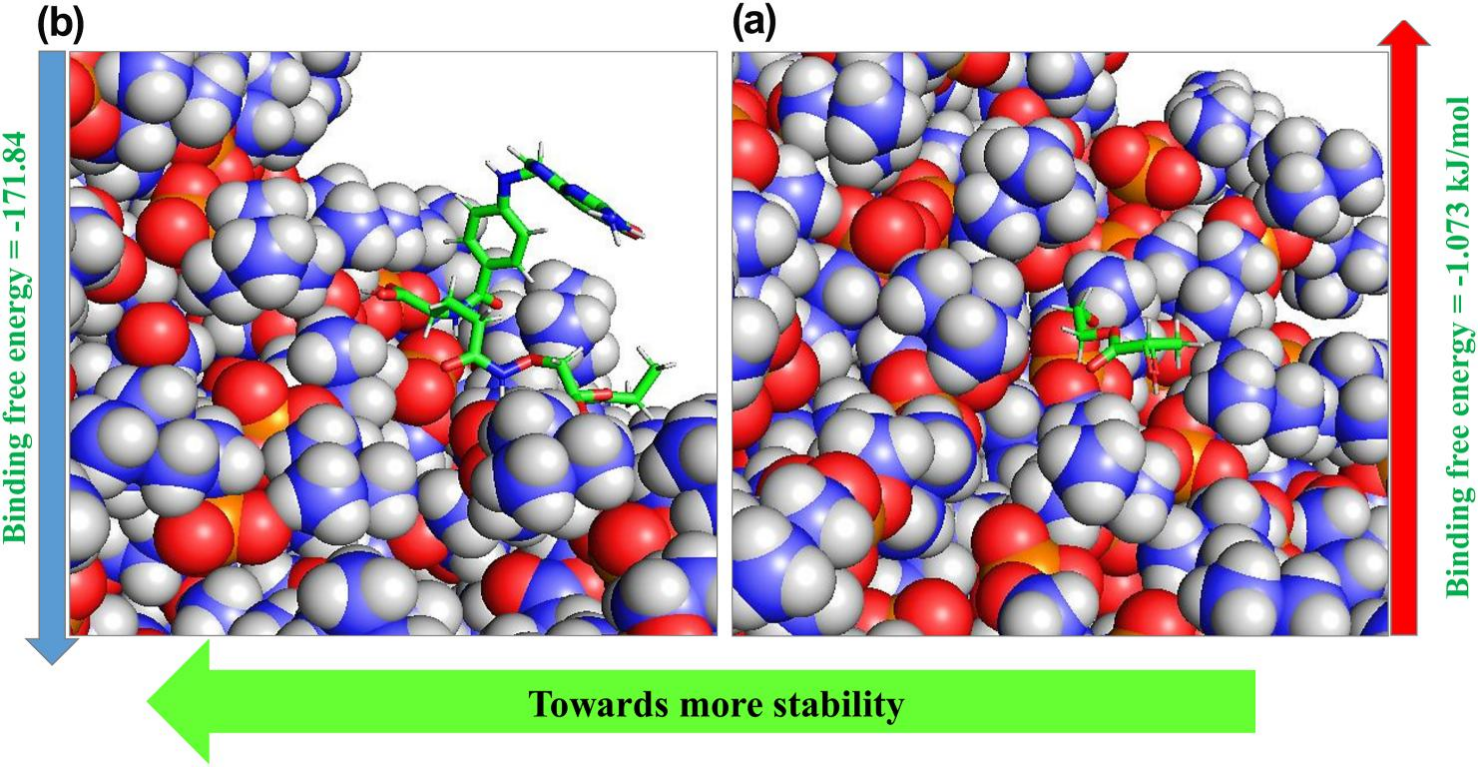
Representation of binding free energy for (a) DPPC & PLA–PEG and (b) DPPC & PLA–PEG‐FA

Tracking the free energy to enhance our knowledge about the stability of the systems is achieved by analysing their components [44]. In this respect, the binding free energy was examined using g_mmpbsa to investigate the stability of the DPPC‐NMs complexes and their interaction tendency. Consistent with observations during docking and Mindist function of MD simulation, the free energy studies showed that the DPPC membrane (−171.84 kJ/mol) forms a more stable complex with PLA–PEG‐FA compared with the PLA–PEG (−1.073 kJ/mol) (Figure 11).

Decomposition of binding free energy for both systems has shown that except for polar solvation energy, in other terms of energy DPPC & PLA–PEG‐FA complex obtained more optimal amounts of energy (Table 3).

**TABLE 3:** Decomposition of binding free energy (kJ/mol) for both nanomicelles extracted from all frames of molecular dynamics trajectories

NM	Functions				
	VdW‐energy	ES energy	PS energy	SASA energy	Total energy
PLA–PEG‐FA	−90.605	−88.417	19.1139	−11.938	−171.84
PLA–PEG	−3.49	−2.61	5.835	−0.808	−1.073

Abbreviations: ES, electrostatic energy; NM, nanomicelle; PS, polar solvation energy; SASA, Solvent accessible surface area; VdW, Van der Waals energy.

**FIGURE 12:**
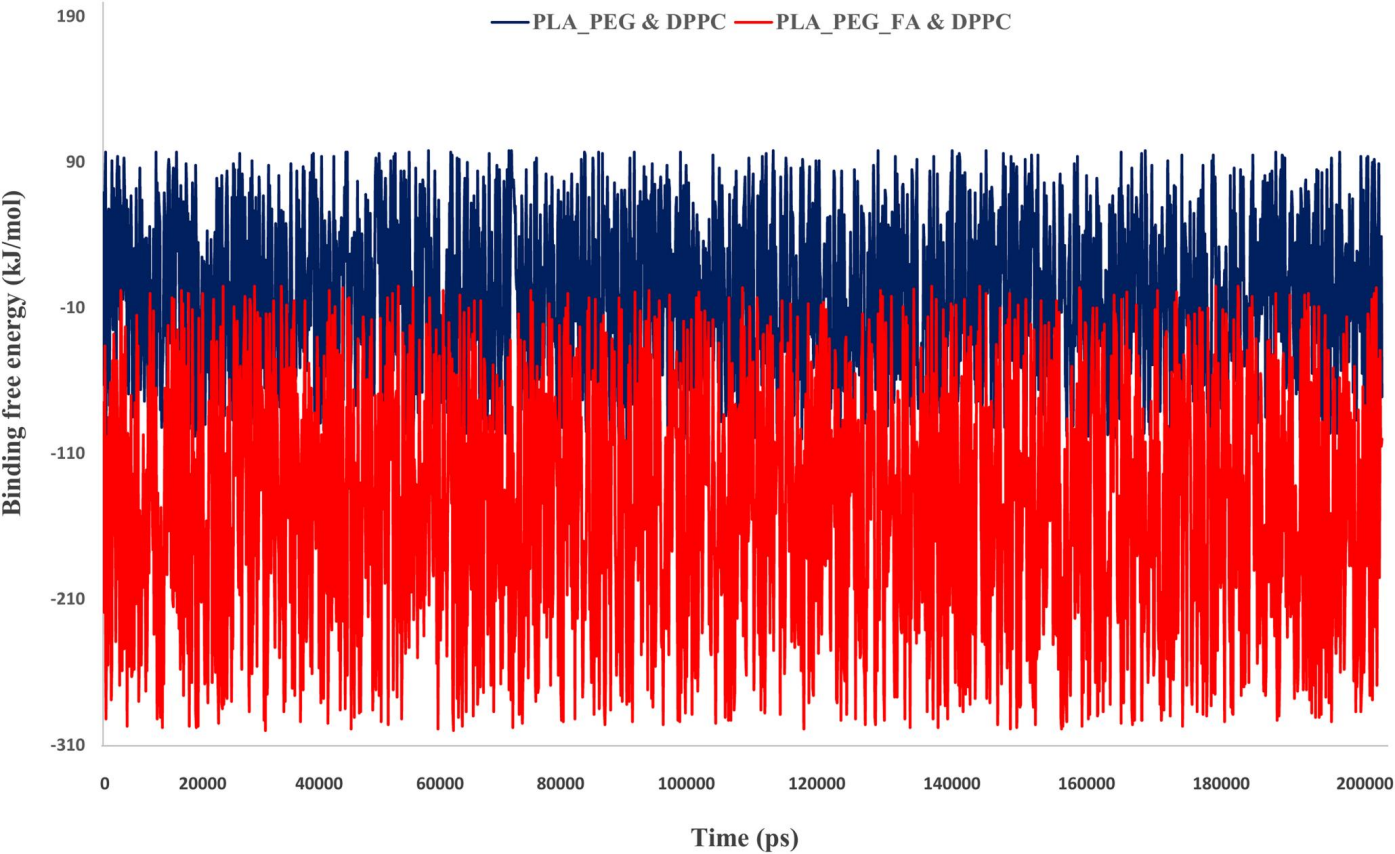
Binding free energy of DPPC complex with designed nanomicelles throughout molecular dynamics

Based on binding (Figure 12) and other terms of free energy obtained at this stage, it can be claimed that the attachment of folic acid to the PLA–PEG, in addition to stabilising the Letrozole‐NM complex, also results in better NM entrapment in the cell membrane.

## 4 CONCLUSION

Targeted NMs offer an effective method against cancer cells. Its main use is to facilitate the penetration of hydrophobic drugs into the cell membrane to increase their effectiveness. In the present study, molecular docking, MD simulations and free energy were used to model the interactions of NMs‐DPPC and letrozole as a widely used aromatase inhibitor. Research shows that folic acid's role is effective in increasing the efficiency of the designed and targeted drug delivery system to the cells of interest. We found that folic acid fused with PLA–PEG increased the stability, penetration and entrapment of Letrozole in the nanoparticle. The explicit and logical results of this study are an essential finding for subsequent studies to differentiate the behaviour of nanoparticles in the vicinity of cell membranes.

## CONFLICT OF INTEREST

The authors declare that there is no conflict of interest and indicate that there are no financial, personal or professional relationships with other people or organisations.

## Data Availability

Data sharing not applicable – no new data generated, or the article describes entirely theoretical research.
